# Interactive Effects of N Form and P Concentration on Growth and Tissue Composition of Hybrid Napier Grass (*Pennisetum purpureum* × *Pennisetum americanum*)

**DOI:** 10.3390/plants9081003

**Published:** 2020-08-07

**Authors:** Chonthicha Pakwan, Arunothai Jampeetong, Hans Brix

**Affiliations:** 1 Department of Biology, Faculty of Science, Chiang Mai University, Mueang, Chiang Mai 50200, Thailand; chonthicha_pa@cmu.ac.th; 2 Research Center in Bioresources for Agriculture, Industry and Medicine, Faculty of Science, Chiang Mai University, Chiang Mai 50200, Thailand; 3 Department of Biology, Aarhus University, 8000 Aarhus C, Denmark; hans.brix@au.dk

**Keywords:** ammonium, energy crop, nitrate, N–P interactions, *Pennisetum*

## Abstract

This study aimed to assess effect of nitrogen (N) form and phosphorus (P) level on the growth and mineral composition of hybrid Napier grass. Experimental plants were grown with different N forms (NO_3_^−^, NH_4_NO_3_, and NH_4_^+^; 500 µM) and P concentrations (100 and 500 µM) under greenhouse conditions for 42 days. Growth rate, morphology, pigments, and mineral nutrients in the plant tissue were analysed. At the low P concentration, the better growth was found in the plants supplied with NH_4_^+^ (relative growth rate (RGR) = 0.05 g·g^−1^·d^−^^1^), but at the high P concentration, the NH_4_^+^-fed plants had 37% lower growth rates and shorter roots and stems. At the high P level, the NH_4_NO_3_^−^-fed plants had the highest RGR (0.04 g·g^−1^·d^−1^). The mineral nutrient concentrations in the plant tissues were only slightly affected by N form and P concentration, although the P concentrations in the plant tissue of the NO_3_^−^-fed plants supplied with the high P concentration was 26% higher compared to the low P concentration plants. The N concentrations in the plant tissues did not vary between treatments. The results showed that the optimum N form for the plant growth and biomass productivity of hybrid Napier grass depends on P level. Hybrid Napier grass may be irrigated by treated wastewater containing high concentrations of N and P, but future studies are needed to evaluate biomass production and composition when irrigating with real wastewater from animal farms.

## 1. Introduction

The discharge of poorly treated wastewater from animal farms has increased with economic growth, particularly in developing countries where wastewater management is poor [1]. Nutrients and organic matter are common contaminants that get discharged into ground and water bodies, thus risking aquatic ecosystems and human health. Nitrogen (N) and phosphorus (P) are generally found in high concentrations in dairy and swine wastewater. Ammonium (NH_4_^+^) is the major form of inorganic N usually found at high concentrations in raw wastewater, but NH_4_ can easily transform into nitrate (NO_3_^−^) via nitrification processes [2]. Additionally, the concentration of P is usually high in wastewater from animal farms. Anaerobic digestion and constructed wetlands (CWs) are conventional methods to reduce the pollutants [3,4,5,6]. However, effluent from the anaerobic digestion still contains high concentrations of N and P that can cause water eutrophication [7,8].

At present, sustainable water use in agriculture has attracted great interest. The reuse of water and nutrients from wastewater through the irrigation of crops by treated wastewater has been identified as a way to mitigate water shortage and, at the same time, recover nutrients as fertilizers. Irrigation with wastewater can improve soil properties, increase plant productivity, reduce the costs of commercial fertilizers and water supplies, and minimize the discharge of nutrients to freshwater ecosystems [9,10,11]. Recycling N and P from wastewater has been applied to many grasses and crops. A study by Leal et al. [12] found that secondary-treated wastewater provided extra elements including N, P, Ca, Mg, and K that could increase the yield of sugar cane (*Saccharum officinarum* L.) compared with a control. Likewise, using secondary-treated abattoir wastewater irrigated crops was found to significantly increase the dry mass yield and nutrient contents of *Pennisetum purpureum* Schumach, *Medicago sativa* L., *Sinapis alba* L., and *Helianthus annuus* L., as well as to increase soil fertility, particularly with N and P [11].

Recently, bioenergy grasses such as switch grass, *Miscanthus*, and Napier grass have been used for removing nutrients from wastewater [13,14,15]. In Thailand, a new hybrid Napier (*Pennisetum purpureum* Schumach × *Pennisetum americanum* (L.) Leeke cv. Pakchong 1) developed by Dr. Krailas Kiyothong, Department of Livestock Development, Ministry of Agriculture and Cooperative, Thailand, has been introduced to the farmers as a fodder crop. Because of its fast growth, disease and drought tolerance, easy propagation, and high yield production, this plant seems to be appropriate as an alternative raw material for biofuel production [16]. Additionally, the Ministry of Energy, Thailand, has paid attention to alternative biofuel sources to replace the use of fossil fuel in electricity generation, and plants with high biomass yield have become targets [17]. As a result, farmers and relevant agencies have encouraged the plantation of this grass. To obtain sufficient material for bioenergy production, the application of fertilizers and water are crucial factors to allow for the high yield of biomass. Recently, a study by Somjai and Suwan [18] found that hybrid Napier Pakchong 1 in an area receiving sufficient water had a higher biomass yield than plants in a non-irrigated area. However, knowledge on fertilizer application, as well as the most efficient way to supply water to the crop field was suggested for further study.

As mentioned above, treated wastewater is a good source of N and P and can be used as an alternative nutrient supply for crop growth on poor soil. However, when reusing wastewater for irrigation, the form of N, as either NH_4_^+^ or NO_3_^−^ or mixed NH_4_NO_3_, and the P concentration may affect plant growth and nutrient acquisition. A previous study found that the hybrid Napier Pakchong 1 grew well when NH_4_^+^ was provided as the major N source [19] and that the plant can tolerate NH_4_^+^ in concentrations up to 5 mM [20]. However, the P concentration used in these studies was rather low (100 µM) compared to the P concentrations in real wastewater. It is therefore not known if the high P levels normally found in real wastewater affect the N acquisition of this species in response to the various N forms interacting with P availability. It has been documented that interactions between N and P availability can promote plant nutrition and increase primary productivity [21,22]. However, plant responses to N and P might be different depending upon concentration levels. Additionally, the differences could be caused by the differences in the uptake mechanism of the two N forms. NO_3_^−^ uptake is an energetically costly mechanism. NO_3_^−^ is taken up into the root cytosol by co-transportation with H^+^ via nitrate transporters (NRTs), while NH_4_^+^ uptake is accompanied by H^+^ extrusion from the root cells. Thus, NO_3_^−^ uptake induces an increase in pH in the rhizosphere, whereas NH_4_^+^ uptake induces a decrease in pH [23]. The changes in pH as a result of N uptake can affect the mobilization of P in the soil and, hence, the P uptake in the plants. Difference ionic charges of NH_4_^+^ and NO_3_^−^ lead to different antagonist or protagonist relationships with P and other ions in the rooting media [24]. It has also been suggested that N–P interactions depend on the supplied N form [25]. To increase our knowledge on this topic, this study aimed to assess the interactive effects of N form and P concentration on growth, morphology, pigments, and mineral composition in the plant tissue of hybrid Napier Pakchong 1. The results from the study will be useful for the development of wastewater management strategies to recycle water and nutrients for agricultural proposes.

## 2. Results

### 2.1. Growth and Morphology

**Figure 1:**
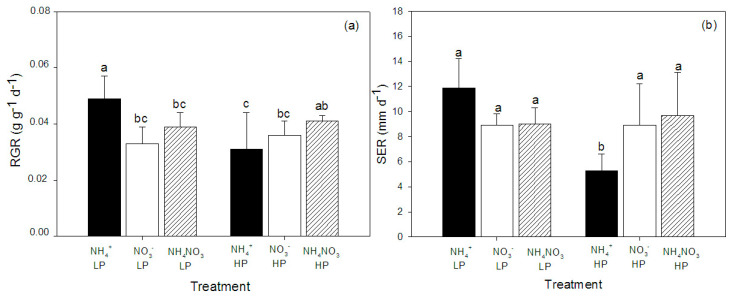
Relative growth rate (RGR) (**a**) and shoot elongation rate (SER) (**b**) of hybrid Napier grass (*Pennisetum purpureum* × *P. americanum*) (mean ± SD) grown on different N forms (NH_4_^+^, NO_3_^−^, and NH_4_NO_3_) and P concentrations (LP, low P concentration (100 µM P); HP, high P concentration (500 µM P)). Different letters above columns indicate significant differences between treatments.

The growth of the hybrid Napier Pakchong 1 was affected by both the N form and P concentration in the growth medium, and significant interactions between the two factors were observed (Table 1). The effects of N form on growth depended on the P concentration in the growth medium, as the lowest relative growth rate (RGR) was found in the plants grown with NH_4_^+^ at a high P concentration, whereas at the low P concentration, the plants grown with NH_4_^+^ had the highest RGR (Figure 1). The RGRs were not significantly different among NO_3_^−^-fed plants and NH_4_NO_3_-fed plants at both the low and high P concentrations. Similarly, the highest shoot elongation rate (SER) was found in NH_4_^+^-fed plants at low P concentrations, and the interaction between these two factors had a significant effect on SERs (Figure 1).

**Figure 2:**
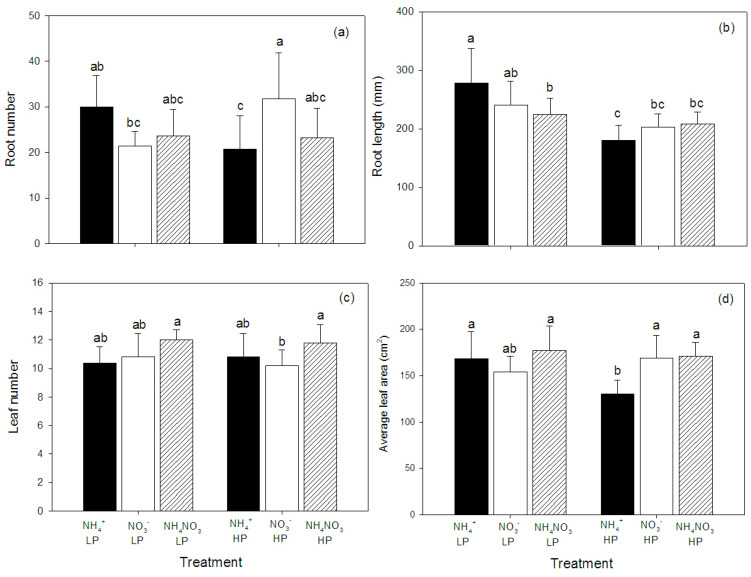
Root number (**a**), root length (**b**), leaf number (**c**), and average leaf area (**d**) of hybrid Napier grass (*Pennisetum purpureum* × *P. americanum*) (mean ± SD) grown on different N forms (NH_4_^+^, NO_3_^−^, and NH_4_NO_3_) and P concentrations (LP, low P concentration (100 µM P); HP, high P concentration (500 µM P)). Different letters above columns indicate significant differences between treatments.

In regard to the plant morphology, the N form and P concentration affected both roots and leaves. As indicated by the significant interactions in the ANOVA (Table 1), the effects of P concentration on the number of roots and root length depended on the N form, as the lowest root number and root length were found in the NH_4_^+^-fed, high P concentration plants, while the plants supplied with NO_3_^−^ at the high P concentration had the highest root number (Figure 2a,b). The leaf areas and number of leaves were highest for the NH_4_NO_3_-fed plants and independent of the P level (Figure 2c,d).

### 2.2. Chlorophylls and Carotenoids

**Figure 3:**
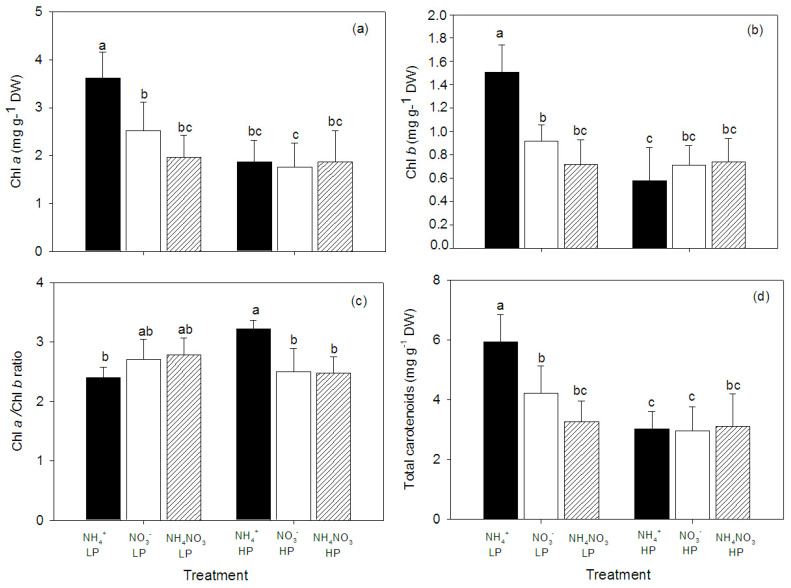
Chlorophyll *a*, Chl *a* (**a**); chlorophyll *b*, Chl *b* (**b**); Chl a/Chl *b* ratio (**c**); and total carotenoids (**d**) in leaves of hybrid Napier grass (*Pennisetum purpureum* × *P. americanum*) (mean ± SD) grown on different N forms (NH_4_^+^, NO_3_^−^, and NH_4_NO_3_) and P concentrations (LP, low P concentration (100 µM P); HP, high P concentration (500 µM P)). Different letters above columns indicate significant differences between treatments.

The concentrations of chlorophyll *a* (Chl *a*), chlorophyll *b* (Chl *b*), and carotenoids were affected by N form and P concentration, and significant interaction between these factors were observed (Table 1). In general, the low P concentration plants had higher contents of chlorophylls and carotenoids than the high P concentration plants. The highest Chl *a* and Chl *b* were found in the NH_4_^+^-fed, low P concentration plants (Figure 3).

### 2.3. Mineral Element Concentrations in the Plant Tissue

**Figure 4:**
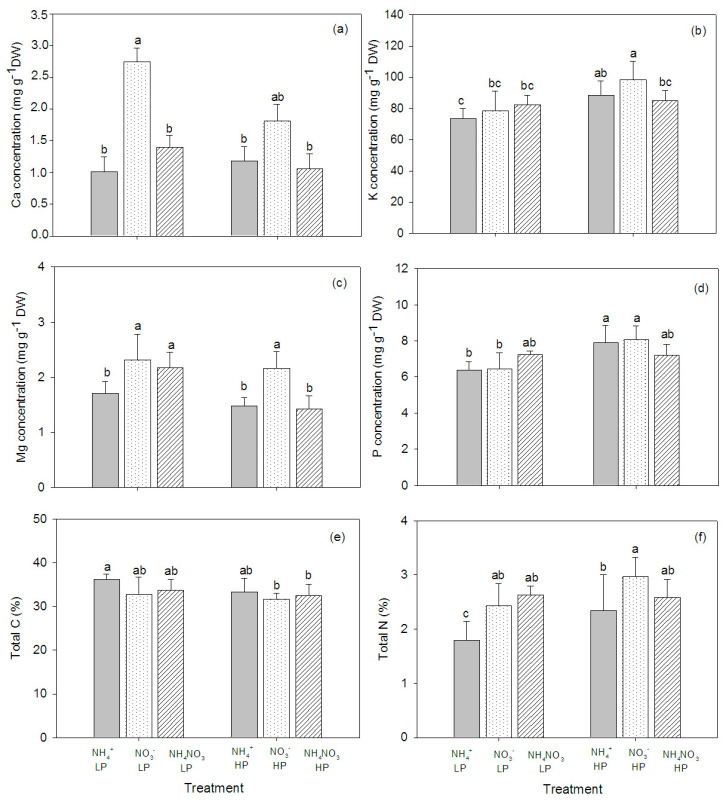
Concentrations of Ca (**a**), K (**b**), Mg (**c**), P (**d**), total C (**e**), and total N (**f**) in stems of hybrid Napier grass (*Pennisetum purpureum* × *P. americanum*) (mean ± SD) grown on different N forms (NH_4_^+^, NO_3_^−^, and NH_4_NO_3_) and P concentrations (LP, low P concentration (100 µM P); HP, high P concentration (500 µM P)). Different letters above columns indicate significant differences between treatments.

**Figure 5:**
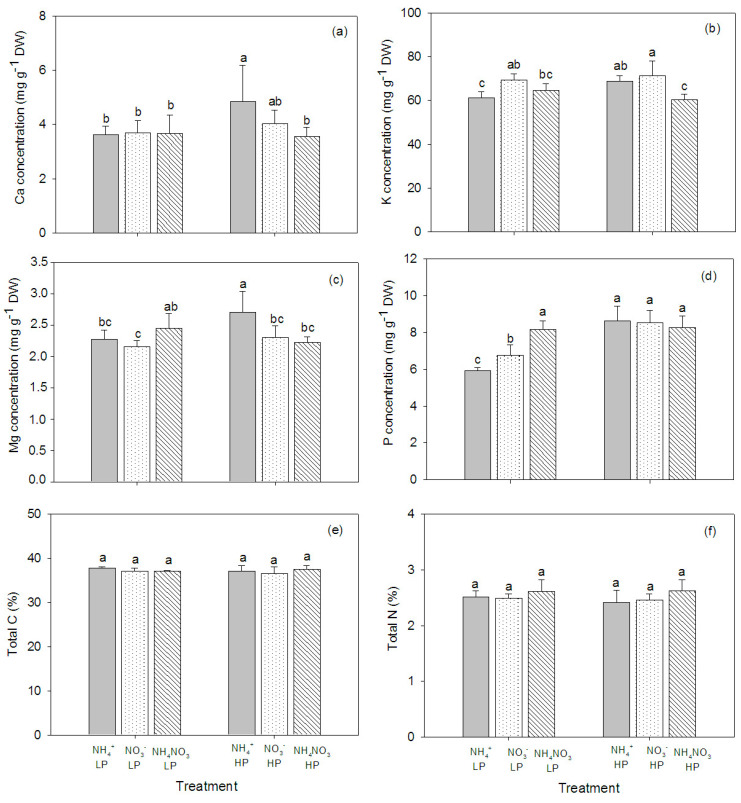
Concentrations of Ca (**a**), K (**b**), Mg (**c**), P (**d**), total C (**e**), and total N (**f**) in leaves of hybrid Napier grass (*Pennisetum purpureum* × *P. americanum*) (mean ± SD) grown on different N forms (NH_4_^+^, NO_3_^−^, and NH_4_NO_3_) and P concentrations (LP, low P concentration (100 µM P); HP, high P concentration (500 µM P)). Different letters above columns indicate significant differences between treatments.

**Figure 6:**
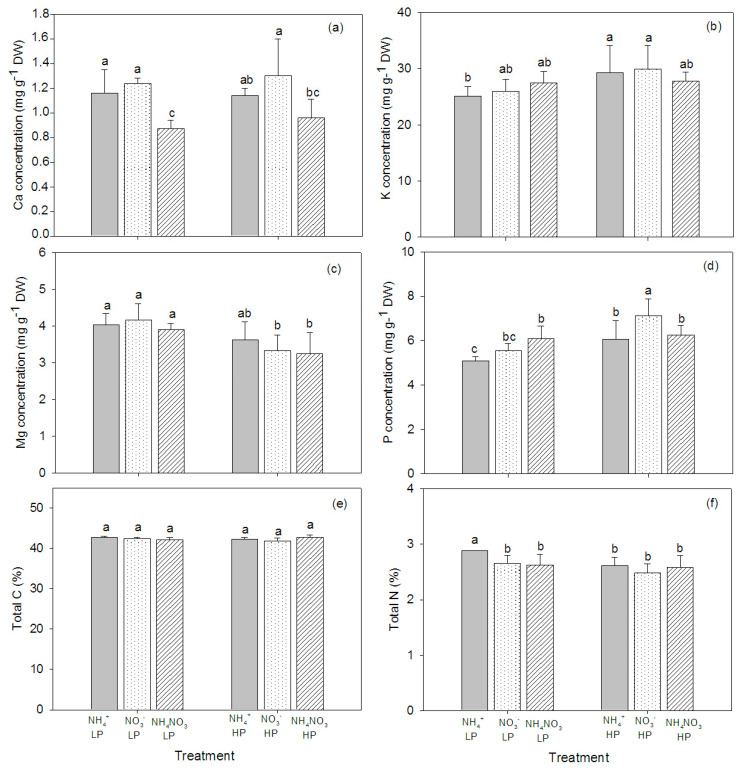
Concentrations of Ca (**a**), K (**b**), Mg (**c**), P (**d**), total C (**e**), and total N (**f**) in roots of hybrid Napier grass (*Pennisetum purpureum* × *P. americanum*) (mean ± SD) grown on different N forms (NH_4_^+^, NO_3_^−^, and NH_4_NO_3_) and P concentrations (LP, low P concentration (100 µM P); HP, high P concentration (500 µM P)). Different letters above columns indicate significant differences between treatments.

N source and P concentration affected the mineral element composition in the plant tissue, and significant interactions between N source and P concentration were observed—particularly in the leaves (Table 2). The concentrations of Ca and Mg in the plant stems were significantly affected by the N form, whereas the P concentration affected the levels of K, Mg, and P in the stems. The NO_3_^−^-fed plants had higher concentrations of Ca and Mg overall, regardless of P concentration. However, K concentration was higher in the high P concentration plants than in the low P concentration plants (Figure 4). In the plant leaves, the concentration of Ca was not affected by the N form and P concentration, but the K concentration increased in NO_3_^−^ fed plants, particularly at the high P supply. P concentration also increased in the plants supplied with the high P concentration (Figure 5). In the roots, Ca was affected by the N form, but K and Mg were affected by the P concentration (Table 2). The highest concentration of Ca was found in the NO_3_^—^fed, high P concentration plants. The concentration of Mg was low in the plants supplied with the high P concentration, while the concentration of K was low in the plants supplied with the low P concentration. Similar to the leaves, P concentration tended to increase in the high P concentration treatments, particularly in NO_3_^−^-fed plants (Figure 6).

### 2.4. CN in the Plant Tissue

The total C content in the plant tissue was not affected by the N form and P concentration. Generally, the total C in roots was higher than in leaves and stems (Figure 4e, Figure 5e, Figure 6e). The total N in the stems was affected by the N form and P concentration (Table 2). The total N slightly increased with the increasing P concentration, while there was no effect on the leaf total N. In roots, the total N significantly decreased in the NH_4_^+^-fed plants exposed to the high P concentration (Figure 4f, Figure 5f, Figure 6f).

## 3. Discussion

N forms and P concentration affected the growth of the hybrid Napier Pakchong 1, and the plants responded to the N form differently depending on P levels. At the low P concentration, the plants grew best when grown on NH_4_^+^, whereas at the high P concentration, the plants performed best with the NH_4_NO_3_ treatment. Previous studies have reported that NH_4_^+^ is the preferred inorganic N form for hybrid Napier Pakchong 1 [19]. However, the current study showed that the N preference depended on the P availability. Other studies have reported interactions between N and P availability on plant growth. Ulrich and Burton [26] found that NO_3_^−^-N and P supply had a strong effect on the growth of *Phragmites australis* (Cav.) Trin. ex Steud., *Sparganium eurycarpum* Engelm., *Typha latifolia* L., and *Typha angustifolia* L., whereas no effect of K was found. The growth of all species fed with high NO_3_^−^ levels were stimulated by increased P additions. Similarly, the growth of *Canna indica* L. increased under the high N and P treatments (6.4 mM as NH_4_NO_3_ and 0.48 mM as KH_2_PO_4_), and interactions between N and P availability were observed. However, the plants supplied with the low N supply (0.36 mM as NH_4_NO_3_) had a low tissue N concentration as a reflection of the N limitation when they were exposed to high P levels [27]. In this study, NH_4_^+^ nutrition interacted with the P level, and synergistic effects on plant growth and N use efficiency were observed. Providing NH_4_^+^ to the rooting medium has been reported to promote P uptake as a result of ammonium-induced acidification in the rhizosphere [25,28,29,30]. However, N uptake can be stimulated at high P concentrations [31]. Likewise, the present study showed that NH_4_^+^ uptake was improved at the high P concentration (data not shown), resulting in an increasing root tissue N concentration. This result was in accordance with the results presented by Romero et al. [32], who found that the NH_4_^+^ uptake rate of *P. australis* increased in the high P concentration (50 µM) treatment. The ability of *P. australis* to adjust the NH_4_^+^ uptake kinetic in response to high NH_4_^+^ and P supply helps the plant to increase its capacity to exploit the excess nutrients and be able to compete with other wetland species. In contrast, in the presence of NO_3_^−^, it is likely that growth and P accumulation in the tissue positively correlates with NO_3_^−^-N when the P supply is high [26]. The hybrid Napier Pakchong 1 fed exclusively with NO_3_^−^-N had a slightly higher RGR at the high P concentration, while the RGR significantly increased in the NH_4_NO_3_ treatment. Moreover, the P concentrations in the tissue, particularly in the roots and leaves, tended to be high in the plants supplied with either NO_3_^−^ or NH_4_NO_3_ at the low P concentration, but significantly higher root P concentrations were found in plants supplied with NO_3_^−^ at the high P level. This might have been related to the fact that ATP is required for active NO_3_^−^ transport across the plasma membrane into root cells. Reduced ATP levels have been reported to directly affect NO_3_^−^ uptake [33,34], confirming the study of Rufty Jr. et al. [35], who found that NO_3_^−^ assimilation and uptake were restricted under P deficiency conditions.

Root morphological adaptations depending on P availability have already been documented [28,36,37]. Root length and root surface area play important roles in excavation and P utilization. P deficiency has been shown to result in increased root length and higher root density [36,38]. In contrast, plants growing at high P concentrations experience a reduction in root length, which is assumed to be a result of P toxicity [37,39]. In this study, the hybrid Napier Pakchong 1 had shorter roots when the plants were grown at the high P concentration. Likewise, the average leaf area and pigment concentrations (chlorophylls and carotenoids) significantly decreased in the plants supplied with the high P concentration, and the strongest effects were found in the NH_4_^+^-fed plants. Leaf chlorosis is a recognized symptom of P toxicity. Many plants such as rice, soy bean, wheat, and *Arabidopsis* show leaf chlorosis when grown under excessive P applications [40,41,42]. Moreover, a study on wild-type rice species (*Oryza sativa* L. “Notohikari”) grown in a hydroponic solution with different P supplies showed a decrease in chlorophylls after being exposed to high P concentration, and the Rubisco activation in the high P-fed plants was also disturbed. As a consequence, the photosynthesis of the plants was inhibited. Similar results found in the study of Shane et al. [43] showed that the photosynthetic rates of *Hakea prostrata* R.Br. significantly decreased after growth at high P supply rates (150 and 300 µmol P d^−1^). In the present study, we found that the NH_4_^+^-fed plants had reduced contents of Chl *a* and Chl *b* (by 48% and 61%, respectively), whereas the leaf P concentrations significantly increased by 45%. This suggested that leaf chlorosis was caused by oxidative stress and photosynthetic disruption. Under a high P accumulation, the Rubisco activity was reduced and caused lower electron sink capacities, thus leading to high electron accumulated in the photosynthetic electron transport chain. Subsequently, the concentrations of reactive oxygen species (ROS) increased. As a result, it can be said that high ROS accumulation triggers leaf chlorosis [42].

Recycling high nutrient-containing wastewater to crops is an attractive prospect for water management, especially in arid agricultural areas. Due to the rapid growth rate of hybrid Napier grass, its ability to utilize various N forms, and its high tissue protein content, the hybrid Napier Pakchong 1 has been recommended for wastewater treatments [19]. Results from this study revealed that high P concentrations may negatively affect plant growth, root length, average leaf area, and chlorophyll contents, particularly when plants are supplied with NH_4_^+^. Mineral and nutrient contents in the plant tissue were, however, only slightly influenced. A study by Ali et al. [31] found that P application can enhance N uptake in maize (*Zea may* L.), but N uptake was limited at high P concentrations. We also found that the high P supply caused a lowering of the root N concentrations, especially in the NH_4_^+^-fed plants. Moreover, Li et al. [44] revealed that P fertilization can affect the biomass productivity and photosynthetic N use efficiency (PNUE) of two *Larix* species. Different responses to the N and P of these plants resulted from the different physiological traits of the species and the different amounts of nutrients in the soil. The present study found that N supply in the form of NH_4_NO_3_ can alleviate the Napier grass from losing biomass, especially at a high P availability. Hence, reusing a high P concentration wastewater to grow crops could be an additional option to prevent the effect of excess ammonium.

## 4. Materials and Methods

### 4.1. Plant Preparation and Experimental Design

The hybrid Napier grass (*Pennisetum purpureum* Schumach × *P. americanum* (L.) Leeke cv. Pakchong 1) used in the experiment was prepared from plant stocks obtained from the Faculty of Fisheries Technology and Aquatic Resources, Maejo University, Thailand. Plant stocks with at least two nodes were selected and placed in trays filled with tap water. After new shoots were produced from the nodes, all plant stocks were supplied with a standard N and P free growth medium prepared according to Smart and Barko [45]. The growth medium had the following composition (mM): K 0.16; Ca 0.62; Mg 0.28; Na 0.69; SO_4_ 0.84; Cl 1.25, B 0.23; Cu 0.02, Fe 0.28; Mn 0.11, Mo 0.006; and Zn 0.04. The pH of the growth medium was adjusted to 6.5. All new plants were propagated under greenhouse conditions at the Department of Biology, Faculty of Science, Chiang Mai University, Thailand.

Approximately 14 days after new shoot were produced, plants of uniform size were selected and separated from the mother stems for use in the experiment. The experimental set-up was a 3 × 2 factorial design with three N forms (NO_3_^−^, NH_4_NO_3_, and NH_4_^+^) at equimolar concentrations (500 µmol N L^−1^) and two levels of P (100 and 500 µM) prepared from KH_2_PO_4_, with 5 replications for each treatment combination. Each experimental treatment combination was established in a hydroponic culture in 40 L containers placed in a greenhouse with a light regime of approximately 12 h light/12 h dark. The temperatures were 30–37 °C day/24–28 °C night. The growth medium in the containers was changed every three days and continuously aerated using air pumps to maintain efficient mixing. At the beginning of the experiment, 10 plants similar to the experimental plants were selected and weighed. The plants were dried in an oven at 55 °C to a constant weight, and the fresh mass to dry mass ratio was calculated and used for growth calculation.

### 4.2. Plant Harvest and Chemical Analyses

All experimental plants were harvested after 42 days of treatment. Total shoot length, average leaf area, number of leaves and roots, and root length were measured. Then, all plants were fragmented into roots, stems, and leaves and freeze-dried. Plant growth was calculated as the RGR and SER. The RGR, based on total plant dry mass, was calculated following the formula: RGR (g g^−1^ d^−1^) = (ln final dry mass − ln initial dry mass)/days [46]. The shoot elongation rate (mm d^−1^) was calculated from the formula: SER = (final shoot length − initial shoot length)/days. The freeze-dried plant materials were used for plant biochemical analysis.

Chlorophyll a (Chl *a*), chlorophyll b (Chl *b*), total chlorophylls (Chl *a* and *b*), and total carotenoids in freeze-dried leaves (n = 5) were determined according to Lichtenthaler [47]. Approximately 8 mg of ground leaves were extracted with 96% ethanol (8 mL). Then, the absorbance of the extracts was measured using a spectrophotometric method at wavelengths of 470, 648.6, and 664.2 nm.

The concentrations of Ca, K, P, Mg, Mn, Fe, and Na in each plant part (n = 5) were analysed in finely-ground dry plant samples (approximately 250 mg). The element concentrations were analysed using inductively coupled plasma emission spectrometry (Optima 2000 DV, PerkinElmer Instruments Inc., Shelton, CT, USA) after microwave digestion in HNO_3_ and H_2_O_2_. The total C and total N contents in the plant tissue (n = 5) were determined from approximately 2 mg of dry plant samples with a CHN analyser (Na2000, Carlo Erba, Italy).

### 4.3. Statistics

Statistical analysis was performed using Statgraphics Plus version 4.1 (Manugistics, Inc., Rockville, MD, USA). The interactions between the N and P factors were analysed by a two-way ANOVA. Assumptions of normality were tested by Levene’s test. Post hoc HSD tests at a 5% significance level were applied to identify differences among treatments.

## 5. Conclusions

The results of the study showed that N form and P level interactively affect the growth, morphology, and mineral concentrations in the tissue of the hybrid Napier Pakchong 1. The responses to N form were found to depend on P level. At the low P concentration, the plants performed best when fed with NH_4_^+^, but at the high P concentration, the plants had a reduced RGR, root number, root length, and chlorophylls and carotenoids contents when only fed with NH_4_. The irrigation of hybrid Napier Pakchong 1 by treated wastewater may be an attractive technique to increase crop yield for fodder or bioenergy. However, future studies are needed to evaluate biomass production and composition when irrigating with real wastewater from animal farms.

## Figures and Tables

**Table 1:** Results of two-way ANOVA (F-ratios) of growth, morphological characteristics, chlorophylls and carotenoids concentrations in leaves of hybrid Napier grass (*Pennisetum purpureum* × *Pennisetum americanum*) grown on different nitrogen (N) forms (NH_4_^+^, NO_3_^−^, or NH_4_NO_3_) and phosphorus (P) concentrations (100 and 500 µM).

	Main Effects		Interaction
	N Form	P Concentration	N Form × P Concentration
Relative growth rate (g·g^−1^·d^−1^)	1.7	2.7	6.8 **
Shoot elongation rate (mm·d^−1^)	0.3	5.5 *	7.8 **
Root number	0.5	0.01	5.0 *
Root length (cm)	0.4	17.5 ***	4.0 *
Leaf number	3.6 *	0.1	0.4
Average leaf area (cm^2^)	4.6 *	0.7	2.91
Chlorophyll *a* (mg·g^−1^·DW)	6.4 **	19.4 ***	5.9 **
Chlorophyll *b* (mg·g^−1^·DW)	6.2 **	12.7 ***	13.8 ***
Total chlorophylls (mg·g^−1^·DW)	7.3 **	23.9 ***	9.1 **
Total carotenoids (mg·g^−1^·DW)	6.1 **	21.5 ***	6.9 **

* *p* < 0.05; ** *p* < 0.01; *** *p* < 0.001.

**Table 2:** Results of two-way ANOVA (F-ratios) of nutrient minerals in the tissue of hybrid Napier grass (*Pennisetum purpureum* × *P. americanum*) grown on different N forms (NH_4_^+^, NO_3_^−^, or NH_4_NO_3_) and P concentrations (100 and 500 µM).

	Main Effects		Interaction
	N Form	P Concentration	N Form × P Concentration
Stems			
Ca (mg·g^−1^ DW)	4.6 *	1.1	0.8
K (mg·g^−1^ DW)	1.5	12.6 **	2.1
Mg (mg·g^−1^ DW)	12.9 **	11.0 **	3.1
P (mg·g^−1^ DW)	0.1	16.0 ***	4.0 *
Total C (%)	2.3	3.0	0.4
Total N (%)	6.9 **	5.1 *	1.6
Leaves			
Ca (mg·g^−1^ DW)	2.1	3.8	2.4
K (mg·g^−1^ DW)	11.4 ***	1.8	5.9 **
Mg (mg g^−1^ DW)	4.3 *	2.4	6.5 **
P (mg·g^−1^ DW)	6.7 **	50.5 ***	12.7 ***
Total C (%)	1.8	0.5	0.7
Total N (%)	1.3	1.0	1.1
Roots			
Ca (mg·g^−1^ DW)	13.8 ***	0.6	0.4
K (mg·g^−1^ DW)	0.4	4.9 *	1.1
Mg (mg·g^−1^ DW)	0.9	16.9 ***	0.6
P (mg·g^−1^ DW)	5.2 *	20.0 ***	3.9 *
Total C (%)	3.3	7.6 *	1.1
Total N (%)	1.7	0.4	3.6 *

* *p* < 0.05; ** *p* < 0.01; *** *p* < 0.001.
